# Efficient genome monomer higher-order structure annotation and identification using the GRMhor algorithm

**DOI:** 10.1093/bioadv/vbae191

**Published:** 2024-11-28

**Authors:** Matko Glunčić, Domjan Barić, Vladimir Paar

**Affiliations:** Faculty of Science, University of Zagreb, Zagreb 10000, Croatia; Faculty of Science, University of Zagreb, Zagreb 10000, Croatia; Faculty of Science, University of Zagreb, Zagreb 10000, Croatia; Department of Mathematical, Physical and Chemical Sciences, Croatian Academy of Sciences and Arts, Zagreb 10000, Croatia

## Abstract

**Motivation:**

Tandem monomeric units, integral components of eukaryotic genomes, form higher-order repeat (HOR) structures that play crucial roles in maintaining chromosome integrity and regulating gene expression and protein abundance. Given their significant influence on processes such as evolution, chromosome segregation, and disease, developing a sensitive and automated tool for identifying HORs across diverse genomic sequences is essential.

**Results:**

In this study, we applied the GRMhor (Global Repeat Map hor) algorithm to analyse the centromeric region of chromosome 20 in three individual human genomes, as well as in the centromeric regions of three higher primates. In all three human genomes, we identified six distinct HOR arrays, which revealed significantly greater differences in the number of canonical and variant copies, as well as in their overall structure, than would be expected given the 99.9% genetic similarity among humans. Furthermore, our analysis of higher primate genomes, which revealed entirely different HOR sequences, indicates a much larger genomic divergence between humans and higher primates than previously recognized. These results underscore the suitability of the GRMhor algorithm for studying specificities in individual genomes, particularly those involving repetitive monomers in centromere structure, which is essential for proper chromosome segregation during cell division, while also highlighting its utility in exploring centromere evolution and other repetitive genomic regions.

**Availability and implementation:**

Source code and example binaries freely available for download at github.com/gluncic/GRM2023.

## 1 Introduction

**Figure 1.**
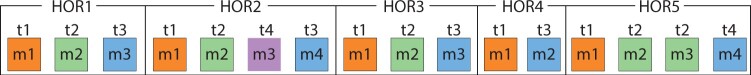
Schematic representation of a monomer array and HORs. Each monomer is represented by a single square. Monomers within HOR unit are labeled as m1, m2, …, in order of their appearance (from left to right within each HOR). Monomers exhibiting <5% sequence divergence are depicted in the same color and labeled with the same identifier (t1, t2,…). A group of three monomers is sequentially repeated to form a higher-order structure known as a 3mer canonical HOR. HOR2, HOR4, and HOR5 are variant HORs due to the insertion (monomer t4 in HOR2, monomer t2 in HOR5) and deletion (monomer t2 in HOR4) of one monomer.

Monomer arrays are composed of primary repeat units, which consist of divergent monomers arranged in a head-to-tail configuration. Individual monomers exhibit a sequence divergence of 2%0–40%. However, the majority of monomers are organized hierarchically into higher-order repeats, secondary repeat units, in which the monomers repeat as structures with high sequence identity (>95%) (Willard and Waye 1987, Choo *et al.* 1991, Willard 1991, Warburton and Willard 1996, Alexandrov *et al.* 2001, Garrido-Ramos 2017, Sullivan *et al.* 2017, Wlodzimierz *et al.* 2023). As depicted in Fig. 1, within a single HOR, all monomers exhibit a variation of 20%–40%, whereas corresponding pairs of monomers across different HORs display less than 5% variation.

The most prevalent HOR copy with *n* constituting monomers is termed canonical *n*mer HOR (3mer HOR in Fig. 1). HOR units within the same HOR array that contain inserts or deletions compared to the canonical HOR unit are known as variants HOR units (for instance, HOR2 with t4 insertion and HOR4 with t2 deletion in Fig. 1).

In this article, we introduce two basic types of HORs: (i) Willard's HORs, where monomers of different types are found within each HOR copy (HOR1, HOR2, HOR3, and HOR4 in Fig. 1). (ii) Cascading HORs, where specific monomer types are reiterated within a canonical HOR copy (HOR5 in Fig. 1).

Monomer HORs in human and nonhuman primates were initially identified through hybridization techniques (Willard 1985, Jorgensen *et al.* 1986, Tyler-Smith and Brown 1987, Willard and Waye 1987, Alexandrov *et al.* 1991, Willard 1991, Warburton and Willard 1996), and subsequently by bioinformatics tools. While various existing software applications effectively identify regions with tandem repeats (Smit et al. 1996–2010, Benson 1999, Novák *et al.* 2010, 2013, 2017, Katoh and Standley 2013, Schaper *et al.* 2015, Sevim *et al.* 2016, Flynn *et al.* 2020), they fall short of providing precise annotations for individual repeat locations or HORs. Similarly, more recent tools designed for annotating human HORs within genomic sequences (Sevim *et al.* 2016, Bzikadze and Pevzner 2020, Dvorkina *et al.* 2020, 2021, Kunyavskaya *et al.* 2022, Gao *et al.* 2023) have limited broader applicability (Wlodzimierz *et al.* 2023). On the other hand, a specific set of software has been developed for the accurate identification of Willard's type HORs (Paar *et al.* 2007, 2011, Gluncic and Paar 2013, Wlodzimierz *et al.* 2023). In the context of the complete assembly of human chromosomes, alpha satellite HORs were initially computed using the NTRprism algorithm (Altemose *et al.* 2022), which bears resemblance to the 2007 version of GRM (Paar *et al.* 2007).

**Figure 2.**
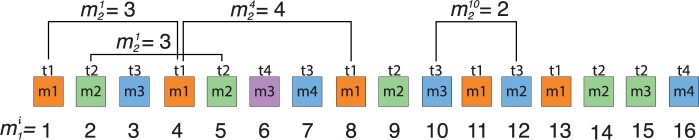
Scheme of an example monomer sequence with 16 monomers, illustrating the first step of the algorithm. The array M consists of 16 2D vectors, $M=\left\{\left(1,3\right),\left(2,3\right),\left(3,4\right),\left(4,4\right),\left(5,4\right),\left(6,0\right),\left(7,3\right),\left(8,3\right),\left(9,5\right),\left(10,2\right),\left(11,2\right),\left(12,4\right),\;\left(13,0\right),\;\left(14,1\right),\;\left(15,0\right),\left(16,0\right)\right\}$. Each monomer is represented by a single square. Monomers within HOR unit are labeled as m1, m2, …, in order of their appearance (from left to right within each HOR). Monomers exhibiting <5% sequence divergence are depicted in the same color and labeled with the same identifier (t1, t2,…).

Here, we introduce our novel GRMhor algorithm and its accompanying application, designed to identify all HORs, including both canonical and variant types, as well as Willard's and Cascading HORs, within monomeric tandem sequences, and graphically display them in the form of diagrams (see Fig. 2) and aligned schemes. The algorithm consists of three complementary components: the GRM diagram, which is based on the concept of the traditional Southern blotting molecular biology technique extended to the monomeric space; the Monomer Distance diagram (MD diagram), which precisely depicts the spatial distribution of periods of monomeric repetitions within the monomeric array; and a aligned schematic representation of HORs array, providing an in-depth visualization of the organization and arrangement of monomers within sequences into HOR structures.

In this study, we present the results of using the GRMhor algorithm to analyse alpha satellite monomers in three individual human chromosome 20 genomes, as well as in the chromosome 20 genomes of three primates: chimpanzee, gorilla, and orangutan. We will demonstrate the significance of this algorithm in the field of individual genomics and evolutionary genomics. Furthermore, in the Section 4, we provide insights into its application on the Neuroblastoma Break Family monomers as reported in our referenced articles (Gluncic *et al.* 2023a, 2023b). Notably, the GRMhor algorithm demonstrates equal efficacy in identifying and analysing HOR structures of any type of monomer across various genomic sequences.

## 2 Methods

In parallel, we will describe the working principles of all three parts of algorithm as they are integrated into a single GRMhor application that utilizes the same input data. Throughout the text, our focus will be on alpha satellite monomers, although the algorithm and application perform equally well for any monomeric repetitions.

### 2.1Algorithm outline

In the first step, we construct an N-dimensional array, $M=\left\{m^{i},\;\ldots ,m^{N}\right\}$, consisting of 2D vectors

(1) $$m^{i}=\left(m_{1}^{i},m_{2}^{i}\right),\;i\in \left[1,N\right]$$

where N is the length of the input monomeric array. The first component of each vector in the array represents the monomer's position in the sequence $\left(m_{1}^{i}=i\right)$, while the second component represents the distance of the monomer at position i to the first adjacent monomer in the sequence that differs from it by less than 5% $\left(m_{2}^{i}=\mathrm{position}\;\mathrm{of}\;\mathrm{first}\;\mathrm{similar}\;\mathrm{monomer}-i\right)$ (Fig. 2). Similarities (differences) between monomers are calculated using the Edlib (Sosic and Sikic 2017) algorithm. For example, if we consider the ith monomer and find that the i+1, i+2, and i+3 monomers differ from it by more than 5%, but the i+4 monomer differs by less then 5%, then $m_{2}^{i}=i+4-i=4$.

The selection of a 5% divergence threshold in the GRMhor algorithm is grounded in the well-documented homogeneity of alpha satellite HORs, as established in numerous studies. For example, (Willard and Waye 1987) demonstrated that HOR units show divergence as low as 1%–5% within arrays, a pattern confirmed by more recent studies such as those by Choo *et al.* (1991) and McNulty and Sullivan (2018), which observed a consistent 95%–99% sequence identity between HOR copies. Furthermore, Alexandrov *et al.* (2001) emphasized the role of sequence homogenization in maintaining this high degree of identity across both ancient and recently evolved HOR arrays

(2) $$p^{j}=\left(p_{1}^{j},p_{2}^{j}\right),\;j\in \left[1,L\right]$$

where L is the maximum distance between any two similar monomers (differs <5%). The first component of the new vector represents the distance between two similar monomers ($p_{1}^{j}=j,\;j=1,\ldots ,L)$, while the second component represents the frequency of occurrence of this distance in the N-dimensional array M, $p_{2}^{j}=\sum_{i=1}^{N}\delta \left(p_{1}^{j},j\right)$, where $\delta \left(p_{1}^{j},j\right)\;$represents the delta function.

In the third step, using the array M, we form groups of monomers such that each group contains monomers differing from each other by less than 5%, and assign each group a name starting from the first, m1, to the last, mk. This way, each monomer in the group is assigned a name, thereby determining its position in the scheme of structural monomer distribution in the third algorithm.

Finally, utilizing the data obtained from the previous steps, we generate two graphs and a schematic representation: (i) the GRM diagram, where we plot the repeat period of monomers, $p_{1}^{j}$ as a function of the frequency of occurrence of each repeat period in the monomeric sequence, $p_{2}^{j}$; (ii) the MD diagram, where we plot the ordinal number of monomers in the sequence, $m_{1}^{i}$ as a function of the distance to the first similar monomer in the sequence, $m_{2}^{i}$; (iii) a aligned schematic representation of the organization of monomers in the sequence, where all monomers from the same group in step three are placed in the same column, sharing the same *x*-coordinate. In the graphical representation, these monomers are depicted by squares of the same color. The squares (monomers) are arranged from left to right and top to bottom according to their appearance index in the sequence, $m_{1}^{i}$, with the condition that when a monomer from the same group appears in the same row, its *y*-coordinate is increased by one, causing it to move to a new row to ensure placement in a column with monomers from its group.

### 2.2 Application usage and output

The input data for our algorithm consists of a series of tandem monomers, which can be obtained in various ways. For the case study presented in the following text, we employed our MonFinder tool (github.com/gluncic/GRM2023), which takes genomic sequences (subject) and consensus sequence (query) as input and delivers a list of detected monomers. This algorithm utilizes the Edlib open-source C/C++ library for precise pairwise sequence alignment (Sosic and Sikic 2017). Within the MonFinder algorithm, the subject sequence is searched in both the direct and reverse complement directions to identify all monomers. In this study, a unique consensus sequence of 171 base pairs (bp) in length (the consensus sequence is located within the MonFinder code on GitHub), derived from over 1 000 000 different alpha satellites across all higher primates, including humans, was utilized as a query for detecting all alpha satellites in the genomic sequence under investigation. In a similar manner, a variety of different tools can be utilized, for instance BLASTN algorithm (Altschul *et al.* 1990).

**Figure 3.**
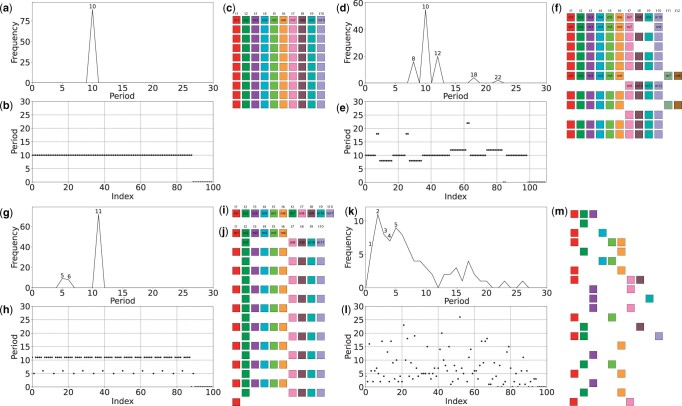
GRM diagrams (a, d, g, k), MD diagrams (b, e, h, l), and higher-order repeat (HOR) schemes (c, f, j, k) for artificial monomers sequences. (a–c) *Willard’s canonical alpha satellite HORs*. (d–f) Willard’s canonical and variant HORs (n = 12, τ = 12) (12 monomers of 12 different types). (g–j) Cascading alpha satellite canonical HORs (n = 10, τ = 11) (10 monomers of 11 different types). (k–m) Randomly distributed alpha satellite monomers. Period denotes the distance between two similar monomers in monomer units. Index denotes the ordinal number of the monomer in the monomeric array. Monomers within HOR unit are labeled as *m*1, *m*2, … *mn*, in order of their appearance from left to right within each row and from top to bottom. Each monomer is depicted by a colored box, with distinct colors corresponding to different monomer types. Monomers are organized into columns based on their monomer types: monomer type *t*1 in the first column, monomer type *t*2 in the second column, and so forth. The number of columns, that is, the number of different monomer types in the canonical HOR unit, is denoted by τ.

The Python program GRMhor (github.com/gluncic/GRM2023) is executed with a file containing a sequence of monomers as the input parameter and optional additional parameters such as the starting monomer in the sequence (default = 0), the maximum value of the displayed period (default = 60), and printing the genomic position of the first monomer in the HOR. After loading the monomer array, the application autonomously proceeds through the steps described in the Algorithm outline, ultimately generating a GRM diagram, MD diagram, and aligned schematic representation of the monomer organization in the array of monomers (Fig. 3). Each generated visualization is automatically saved in three distinct .ps files in the initial directory. In the following chapter, through several case studies, first with artificial arrays of monomers, and then with monomers from real sequences of the human and higher primates genomes, we will elucidate how to interpret each of these visualizations and easily identify and analyse HORs types, organization and structure in detail.

## 3 Results

### 3.1 Algorithm

In the following four artificial case studies, we utilized actual monomers from of the T2T-CHM13v2.0 assembly, selecting 10 distinct alpha satellites (with a mutual difference >20%), to construct various artificial monomer arrays. All artificial monomer arrays are available for testing on github.com/gluncic/GRM2023. Each of these artificial arrays was then subjected to our algorithm, with a detailed discussion of the results provided. Subsequently, we conducted an analysis of the entire real sequences of the three individual human chromosome 20 assemblies and three higher primates chromosome 20 assemblies.

#### 3.1. 1 Willard’s canonical alpha satellite HORs

We replicated a set of 10 distinct monomers 10 times, resulting in a sequence of 100 monomers, where each monomer possesses ten identical copies. In the GRM diagram (Fig. 3a), a distinct peak corresponding to a period of ten is observed, providing clear evidence that all similar monomers are spaced at a distance of 10 monomers from each other. The same conclusion can be drawn from the MD diagram (Fig. 3b), where each point represents a vector $\left(m_{1}^{i},m_{2}^{i}\right)$, namely a function of the monomer's position in the sequence and the distance to the first similar monomer. All points lie on the ordinate *y* = 10, indicating that any two similar monomers are situated at a distance of 10 monomers in the monomeric array. Together, we can conclude that our monomers form a 10-order HOR, that is, a 10mer HOR, as also evident from the schematic representation of the organization of monomers in Fig. 3c. To facilitate the description of more complex structures in the following case studies, we will introduce two distinct labels for monomers within the HOR unit (Fig. 3c). With the label *t*τ, we will denote all similar monomers at position τ within the HOR unit, while with the label mn, we will denote the ordinal number of the monomer within the HOR unit. In Willard-type HORs, the labels of these two designations for each monomer within the HOR unit are identical (τ=n) (Fig. 3c). In the MD diagram, the last 10 monomers (Index 91–100) exhibit a period of zero as none of them finds a similar monomer to the end of the sequence.

#### 3.1. 2 Willard’s canonical and variant alpha satellite HORs

In the sequence of 100 monomers from the previous case study (Section 3.1.1), we made modifications by deleting the 18th, 19th, 38th, and 39th monomers and inserting two new monomers (distinct from the initial 10) after the 66th and 86th monomers (see Fig. 3f). This was done to simulate variant Willard’s HORs with deletions and insertions. The dominant peak on the GRM diagram (Fig. 3d) remains at a period of 10, albeit with a slightly lower frequency. New peaks emerge at periods, in order of frequency, 12, 8, 18, and 22. In the MD diagram (Fig. 3e), alongside the highest concentration of points distributed at *y* = 10, new sequences of points also appear at the corresponding new periods.

The peaks at periods 8 and 18 correlate with an additional set of points on the left side of the MD diagram, indicating that these periods result from the emergence of new HOR variants through the deletion of monomers. Figure 3f reveals that the first seven monomers in the second, variant HOR (second row in Fig. 3f) now repeat not after 10, but after eight monomers, due to the absence of the deleted monomers t7 and t8 in this HOR. The same pattern is observed with the fourth, variant HOR unit. Consequently, two sets of eight points at period *y* = 8 appear on the MD diagram. Furthermore, monomers t7 and t8 in the first, canonical HOR unit lack similar copies in the second, variant HOR unit, and their similar copies are only found in the third, canonical HOR unit, repeating after 8 + 10 = 18 monomers. A similar scenario applies to monomers t7 and t8 in the third, canonical HOR unit. Consequently, two sets of two points at period *y* = 18 appear on the MD diagram.

The peaks at periods 18 and 22 in the GRM diagram and the series of points at the same ordinates in the MD diagram are the result of the insertion of two new monomers in variant HOR units. It is evident that in these HOR units, due to the two additional monomers, the first six monomers are repeated only after 12 monomers. Additionally, the first pair of two additional monomers finds its similar monomers only after a sequence of 5 + 10 + 7 = 22 monomers. The second pair of two monomers does not find similar monomers until the end of the sequence; therefore, in the MD diagram, we only have one set of two points at a *y* = 22.

In conclusion, it is quite straightforward from the GRM diagram and the MD diagram to conclude that, in general, we are dealing with a *n*mer canonical HOR of Willard’s type, along with some variant HORs obtained through deletions or insertions of new monomers.

#### 3.1. 3 Cascading alpha satellite canonical HORs

In the sequence of 100 monomers from the previous case study (Section 3.1.1), we made modifications by inserting monomer t2 into each HOR after monomer t6, so that the monomeric sequence in each HOR resembles consensus HOR shown in Fig. 3j. Now, the dominant peak in the GRM diagram is at period 11, with significantly lower peaks at periods 5 and 6 (Fig 3g). Accordingly, the majority of points in the MD diagram are also found at period 11, with fewer at periods 5 and 6 (Fig. 3h). From the scheme in Fig. 3j, it is clear that all monomers, except *m*2, in this situation encounter a similar monomer after 11 monomers. Furthermore, the first copy of *m*2 in each HOR encounters a similar monomer after five other monomers, and the second copy of *m*2 in each HOR encounters a similar monomer in the next HOR after six monomers. This accounts for the peaks at 5 and 6 in the GRM diagram, or the points at *y* = 5 and *y* = 6 in the MD diagram. Altogether, both diagrams clearly indicate an 11mer Cascading HOR with a duplicated single similar monomer.

#### 3.1. 4 Randomly distributed alpha satellite monomers

As our final artificial case study, from an initial sample of 10 distinct monomers, we constructed a series of 100 tandem monomers by duplicating them randomly using Python's default random number generator based on the Mersenne Twister algorithm. Considering that the examined sequence resulted from the random duplication of 10 initial monomers, we anticipate that the distribution of peaks in the GRM diagram will be highest at small periods. Both the GRM diagram (Fig. 3k) and the MD diagram (Fig. 3l) in this instance indicate a lack of higher-order organization, which is further illustrated in the schematic in Fig. 3m.

### 3.2 Implementation

#### 3.2. 1 Case study: alpha satellite monomer HORs in three individual human chromosome 20 genomes

Using our MonFinder algorithm, we have isolated all alpha satellites in three individual human chromosome 20 genomes: the T2T Consortium CHM13v2.0 assembly (GCA_009914755.4) (Nurk *et al.* 2022) and the only two genomes from the Human Pangenome Reference Consortium (UCSC Genomics Institute) sequenced to the chromosome level, HG002v1.0.1 (GCA_018852615.2) (Liao *et al.* 2023) and HG01243v3.0 (GCA_018873775.2) (Zimin *et al.* 2022).

As a result, we identified 24 128 alpha satellites in chromosome 20 of the CHM13 assembly, 27 028 alpha satellites in HG002 chromosome 20 assembly, and 30 096 alpha satellites in HG01243 chromosome 20 assembly, with the majority located in several blocks of tandem repeats.

**Figure 4.**
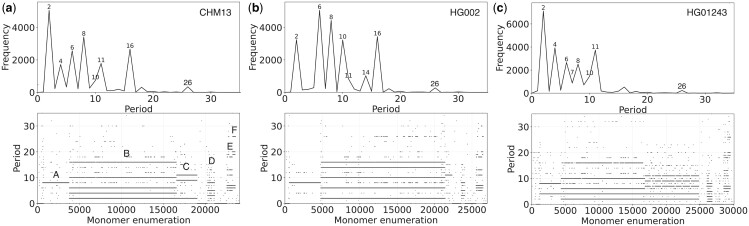
Global repeat map (GRM) diagram and MD diagram for tandemly arranged alpha satellite monomers in the complete assemblies of human chromosome 20: (a) T2T-CHM13v2.0 (GCA_009914755.4), (b) Pangenome Reference Consortium HG002 (GCA_018873775.2), and (c) Pangenome Reference Consortium HG01243 (GCA_018873775.2). GRM diagrams: Horizontal axis: GRM periods (in monomer units). Vertical axis: frequency of monomer repeats period. Identified GRM peaks exhibit periods 2, 4, 6, 8, 10, 11, 16, 18, and 26. The significance of these GRM peaks (HORs or associated subfragment repeats) can be inferred from the monomer distance (MD) diagram. MD diagram: Horizontal axis: enumeration of tandemly organized alpha satellite monomers, in sequential order as revealed by GRM analysis of the assembly. Vertical axis: period (the distance between start of a monomer and of the next monomer of the same type (see Fig. 2)). Four distinct regions of monomer tandems are denoted by A, B, C, D, E, and F. Additionally, there are sporadic MD points that do not correspond to HORs or their subfragments.

From the MD diagrams (lower panels in Fig. 4a–c), we can easily identify six distinct HOR regions in all three genomes. These regions generate various prominent peaks on the GRM diagram (upper panels in Fig. 4a–c). Region A comprises 8mer HORs, region B consists of 16mer HORs, region C contains 11mer HORs, region D encompasses 8mer HORs, region E comprises highly variant 18mer Cascading HORs, and region F contains 26mer Cascading HORs. In regions containing multiple variant HORs, we determine the dominant *n*meric HOR based on the highest number of dots at a specific period in the MD diagram and the most frequent pattern in the schematic representation (Supplementary Figures). A comprehensive schematic representation of the HORs, along with the first monomer positions of HORs within the genomic sequence, is provided in the Supplementary Materials (Supplementary Figs. S1–S17, for regions A–F, respectively). For a clearer presentation of each aligned scheme in Supplementary Figs. S1–S17, individual blocks of monomers were extracted from each region according to the indices in the MD diagram and reprocessed through the GRMhor algorithm.

**Figure 5.**
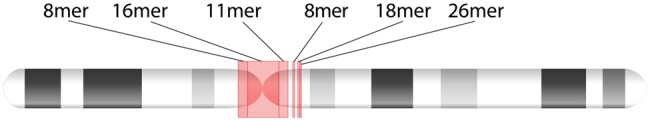
Ideogram of major alpha satellite HOR arrays in the CHM13v2.0 assembly of human chromosome 20.

All these regions are located approximately in the same positions in all three individual genomes and, as shown in Fig. 5, belong to the centromeric and pericentromeric parts of chromosome 20.

**Figure 6.**
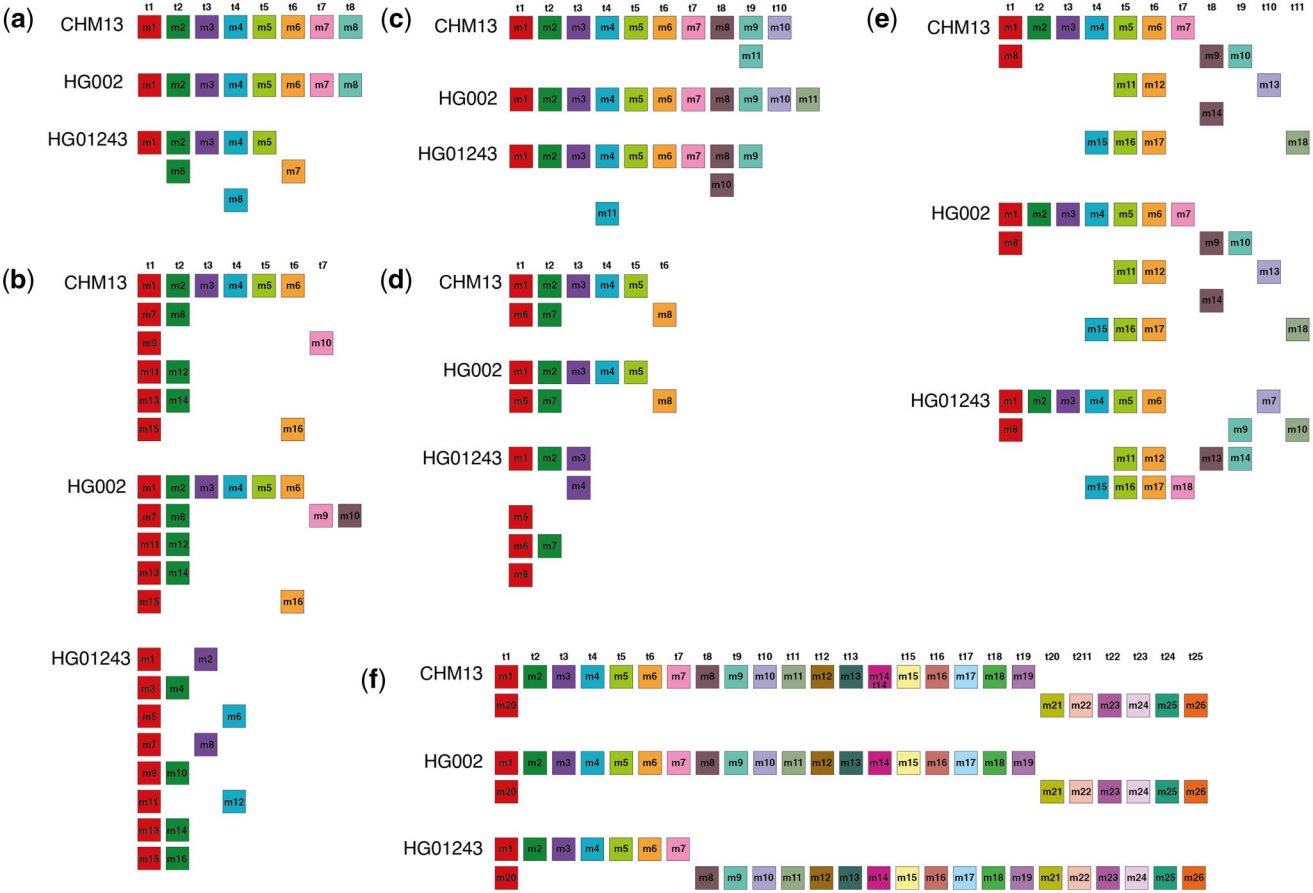
Aligned schemes of the canonical HOR units from all HOR regions in three individual assemblies of human chromosome 20. (a) 8mer HOR in region A. (b) 16mer HOR in region B. (c) 11mer HOR in region C. (d) 8mer HOR in region D. (e) 18mer HOR in region E. (f) 26mer HOR in region F. The first row in each panel shows the corresponding HOR consensus from chromosome 20 of the CHM13 genome, the second row from the HG002 genome, and the third row from the HG01243 genome. Monomers within HOR copy are labeled as m1, m2, …mn, in order of their appearance (from left to right within each row and from top to bottom). Each monomer is depicted by a colored box, with distinct colors corresponding to different monomer types. Monomers are organized into columns based on their monomer types: monomer type t1 in the first column, monomer type t2 in the second column, and so forth. The number of columns, that is, the number of different monomer types in the canonical HOR copy, is denoted by τ.

In Region A (Fig. 4, Supplementary Figs. S1, S7, and S13), the HOR units in the CHM13 and HG002 genomes are predominantly Willard's consensus HORs (Fig. 6a), which are evident from the MD diagram as they generate peak 8 in the GRM diagram. In contrast, the HG01243 genome exhibits a Cascading type of 8mer HOR (Fig. 6a), generating two MD lines at positions 8 and 4, along with equivalent peaks in the GRM diagram. Furthermore, the individual genome HG002 has approximately 25% more total and canonical 8mer HOR units in this pericentromeric region (Table 1).

**Table 1.** Alpha satellite HOR arrays in three individual assemblies of human chromosome 20 determined using GRMhor algorithm.

HOR	No. of mon. types in HOR			No. of HOR copies			No. of canonical HOR copies			Type of HOR		
	hs1	hs2	hs3	hs1	hs2	hs3	hs1	hs2	hs3	hs1	hs2	hs3
8mer	8	8	6	388	513	385	357	493	360	W	W	C
16mer	7	8	4/4	790	1074	1666	637	801	795/575	C	C	C
11mer	10	11	9	235	88	652	197	74	532	C	W	C
8mer	6	6	3	107	66	90	68	47	39	C	C	C
18mer	11	12	10	37	44	36	21	24	8	C	C	C
26mer	25	25	25	14	5	14	13	4	7	C	C	C

The three individual genomes are abbreviated as follows: hs1—CHM13, hs2—HG002, hs3—HG01243. Type of HOR: W—Willard’s, C-cannonical. In the individual genome hs3—HG01243, the 16mer HOR (second row) appears as an alternating combination of a 6mer and a 10mer HORs. Accordingly, in the column “No. of mon. types in HOR” (4/4), the first number refers to the number of distinct monomers in the 6mer, while the second number refers to the number in the 10mer. Similarly, in the column “No. of canonical HOR copies” (795/575), the first number indicates the number of canonical copies of the 6mer, and the second number corresponds to the number of canonical copies of the 10mer.

HOR units in region B cover the entire centromere and are cascading types of 16-mer HORs in all three individual genomes (Fig. 6b, Supplementary Figs. S2, S8, and S14). All consensus HORs are characterized by a large number of duplicated monomers, particularly t1 and t2 types (red and green squares in Fig. 6b). These duplications of two monomers within the same HOR result in peaks at periods 2, 4, and 6. Additionally, the 16mer HOR in the HG01243 genome has a different structure in this region, containing only four distinct types of monomers and therefore consisting of two subfragments with lengths of 6 and 10 monomers (Table 1). The HG002 and HG01243 genomes have approximately 35% and 100% more 16mer HOR units, respectively, compared to the CHM13 genome (Table 1), particularly because some of these HOR units in the HG01243 genome extend into region C, where they alternate with 11mer HORs.

The HOR units in region C are cascading 11mer HORs in the genomes of CHM13 and HG01243, and Willard-type 11mer HORs in the HG002 genome (Fig. 6c and Supplementary Figs. S3, S9, and S14). As previously noted, an additional characteristic of this region in the HG01243 genome is the presence of alternating sequences of 11mer and 16mer HORs (Supplementary Fig. S14), while pure sequences of 11mer units are found in the HG01243 and HG01243 genomes. Nonetheless, the HG01243 genome has a significantly higher number of 11mer HOR units (Table 1), whereas in the HG002 genome, region C and the number of 11mer HORs are the smallest (Fig. 4c, lower panel).

The HOR units in region D of all three individual genomes are cascading 8-mer HORs with duplicated monomers *t*1 and *t*2. Additionally, the consensus 8-mer HOR in this genomic region of HG01243, besides having multiple duplications of *t*1 and *t*2, also includes a duplicated monomer *t*3. Therefore, the HOR in HG01243 consists of only three different types of monomers, while in the genomes CHM13 and HG002, it consists of six different types of monomers (Fig. 6d, Supplementary Figs. S4, S10, and S15). The CHM13 genome contains approximately 40% and 15% more 11-mer HORs than HG002 and HG01243, respectively (Table 1). All three genomes in this region exhibit a high proportion of variant HORs, suggesting that this region is subject to significant mutations or errors during the duplication of HORs in the crossing-over process.

The highly cascading 18mer HOR units in E region, present in all three individual genomes, exhibit significant complexity, featuring five duplicated monomers (Fig. 6e, Supplementary Figs. S5, S11, and S16). The structure of these highly cascading HORs is identical in the CHM13 and HG002 genomes, while the HG01243 genome exhibits a single distinct monomer duplication. HG002 has a slightly higher number of 18mer HOR units, whereas the HG01243 genome has a very large proportion of variant HORs, similar to the previous region (Table 1).

In region F, a complete set of canonical 26mer Cascading HOR units with only one recurring monomer (*t*1) is found (Fig. 6f, Supplementary Figs. S6, S12, and S17). The structure of these HOR units is the same across all three individual genomes. However, in the CHM13 and HG002 genomes, *t*1 monomer repeats after 18 units, whereas in the HG01243 genome, it repeats after 7 units. This region contains a very small number of HOR units. Notably, the HG002 genome has two-thirds fewer 26mer HOR units compared to the other two genomes (Table 1). All 26mer HOR units in the CHM13 genome are canonical, while in the HG01243 genome, half of the HOR units in this region are variant.

The comparison of canonical HORs across individual genomes shows that each of the six identified consensus HORs consists of distinct monomeric units, with only the 11mer and 25mer HORs sharing an overlap of six monomers. Additionally, cross-genome comparison of identical *n*mer HORs reveals that each canonical HOR is constructed from the same monomeric units across genomes. Therefore, we can conclude that key differences lie not in the distinct monomeric units that form the HORs or point mutations within monomers but rather in the structural arrangement of these monomers, the canonical HOR arrangement, the variety and count of monomers, and the distribution of canonical versus variant HOR units within each HOR array (Fig. 6 and Table 1).

The differences observed in HOR units among the genomes CHM13 (hs1), HG002 (hs2), and HG01243 (hs3) are likely attributable to inherent biological variability in tandem repeats. These HORs are dynamic and susceptible to structural rearrangements driven by mechanisms such as unequal crossing-over, gene conversion, and replication slippage, leading to individual-specific expansions and contractions within repeat arrays. While the genomes may share common monomer types, variations in HOR and canonical copy numbers indicate unique evolutionary events that can alter repeat structures.

#### 3.2. 2 Case study: alpha satellite monomer HORs in chromosome 20 genomes of three higher primates

In the following case studies, we examine the alpha satellite structures on chromosome 20 in three different higher primates: chimpanzee (NHGRI_mPanTro3-v2.0_pri GCA_028858775.2), gorilla (NHGRI_mGorGor1-v2.0_pri GCA029281585.2), and orangutan (NHGRI_mPonAbe1-v2.0_pri GCA_028885655.2). We identified 23 164 alpha satellite monomers in the chimpanzee, 46 885 in the gorilla, and 47 709 in the orangutan chromosome 20 genome. Notably, the number of alpha satellites in the gorilla and orangutan chromosome 20 is significantly increased—by over 35%—compared to the chimpanzee and all three individual human genomes discussed in Section 3.2.1.

**Figure 7.**
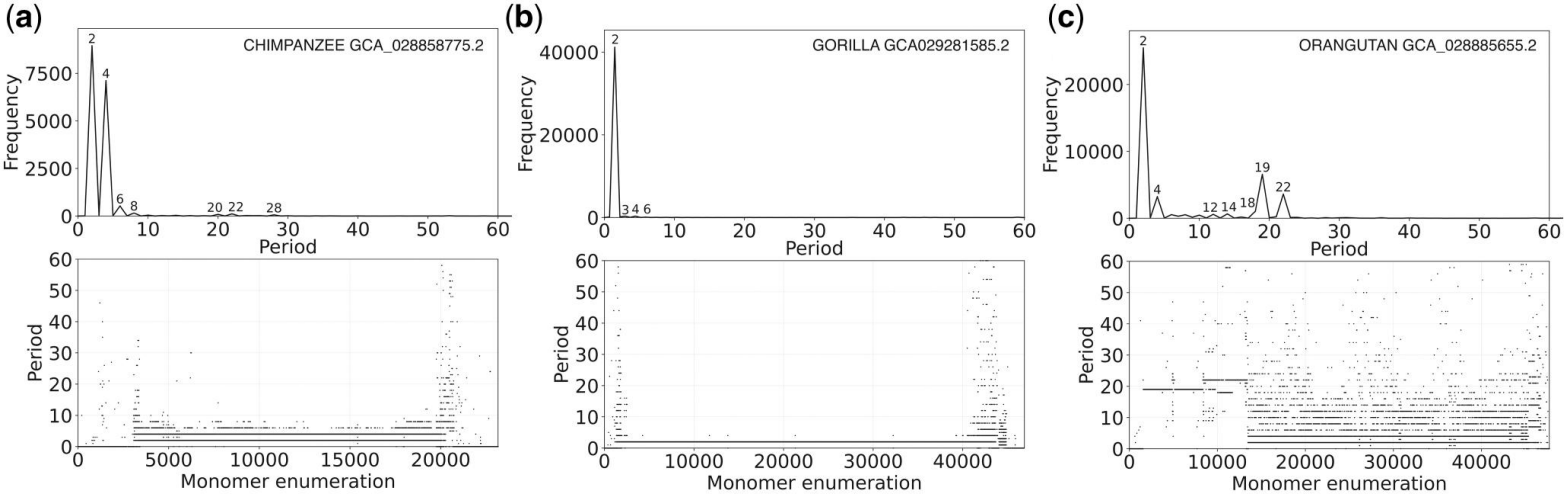
Global repeat map (GRM) diagram and MD diagram for tandemly arranged alpha satellite monomers in the complete assemblies of higher primates: (a) Chimpanzee (GCA_028858775.2), (b) Gorilla (GCA029281585.2), and (c) Orangutan (GCA_028885655.2). GRM diagrams: Horizontal axis: GRM periods (in monomer units). Vertical axis: frequency of monomer repeats period. The significance of identified GRM peaks (HORs or associated subfragment repeats) can be inferred from the monomer distance (MD) diagram. MD diagram: Horizontal axis: enumeration of tandemly organized alpha satellite monomers, in sequential order as revealed by GRM analysis of the assembly. Vertical axis: period (the distance between start of a monomer and of the next monomer of the same type (see Fig. 2)).

From the MD diagrams (lower panels in Fig. 7a–c) and GRM diagrams (upper panels in Fig. 7a–c), we can clearly identify a significantly lower degree of alpha satellite organization into higher-order structures in chromosome 20 across all three great apes compared to the human genome. Chimpanzee and gorilla predominantly exhibit 2mer and 4mer HORs, while in the chromosome 20 genome of orangutan, additional 19mer and 20mer HORs are present.

A more detailed analysis of the aligned schemes in chimpanzees reveals that in the first region, from monomer enumeration approximately 4000–20 000 (Fig. 7a), alpha satellites are exclusively organized into 4mer HORs (Supplementary Fig. S18, Table 2). In the peripheral region, after monomer enumeration 20 000, a small number of 20mer cascading HORs (comprising five canonical HOR units) emerge (Supplementary Fig. S19), followed closely by a small number of 22mer cascading HORs (comprising seven canonical and one variant HOR units) (Supplementary Fig. S20, Table 2). All identified chimpanzee alpha satellite HORs have distinct alpha satellites and higher-order structures compared to the HORs identified in the three individual human genomes shown in Table 1.

**Table 2.** Alpha satellite HOR arrays in three higher primates’ chromosome 20 assemblies determined using GRMhor algorithm.

Species	HOR	No. of mon. types in HOR	No. of HOR copies	No. of canonical HOR copies	Type of HOR
Chimpanzee	4mer	3	4250	3209	Cascading
	20mer	19	5	5	Cascading
	22mer	19	8	7	Cascading
Gorilla	2mer	2	20 620	20 309	Willards
	6mer	4	128	64	Cascading
Orangutan	19mer/22mer	19/22	614	344/172	Willard
	2mer	2	14 633	11 778	Willard
	4mer/6mer/8mer/12mer	4	480	85/50/35/61	Cascading

Similarly, the majority of alpha satellite sequences on the gorilla chromosome 20 do not organize into more complex higher-order structures, but instead appear as simple 2mer HORs (Fig. 7b, Table 2). An exception is a short region at the end of the 2mer array (monomer enumeration approximately 44 000), which consists of a tandem array of 128 6mer HOR units (Supplementary Fig. S21, Table 2).

In the orangutan genome, the situation is somewhat different. A portion of the alpha satellite is organized into 19mer HORs and subsequently into 22mer HORs, which interweave within a specific region of the genome (Fig. 7c, Supplementary Fig. S22). The second, and also the longest, region of the alpha satellite is organized, as in the other two higher primates, into simple 2mer HOR structures (Table 2). Finally, there is a rather irregular region where 4mer (85 HOR units), 6mer (50 HOR units), 8mer (35 HOR units), and 12mer (61 HOR units) HORs are interwoven, along with a large number of various variants of these HORs (Table 2).

In conclusion, unlike in human chromosome 20, where alpha satellite monomers are primarily organized into simple 2mer or 4mer higher-order repeat structures, the genomes of other higher primates exhibit more complex HOR structures at the peripheries, though in fewer copies.

## 4 Discussion

In artificial case studies, we demonstrated that the GRMhor algorithm can effectively detect all types of HOR arrays, whether they are fully canonical or exhibit various variant modifications.

Subsequently, we showed that even in the complex structure of the individual human assembly chromosome 20 assemblies, the GRMhor algorithm successfully identifies alpha satellite HOR arrays and reveals their internal structure. Six distinct HOR arrays were delineated: the Cascading 16mer HOR, the Willard/Cascading 11mer HOR, the Willard/Cascading 8mer HOR, Cascading 8mer HOR, Cascading and almost complete canonical 26mer HOR, and highly Cascading and highly variant 18mer HOR (Table 1).

The study by Altemose *et al.* (2022) corroborates the identification of the same HOR structures as the GRMhor algorithm, particularly 16mer, 8mer, 11mer, 8mer, 18mer, 26mer, and 6mer. The only difference lies in the 6mer HOR, which in GRMhor algorithm, unlike in Altemose *et al.* (2022), does not possess the status of a distinct HOR, as it represents a variant of the 16mer and 18mer HORs, as evident in Fig. 7a and b. In Altemose *et al.* (2022), it is stated that this 6mer HOR is divergent, and the region occupied by its repetitions is very short (19996bp), corresponding to 117 monomers, or 20 variant HORs.

In the computation using HiCAT algorithm, five HORs were reported in chromosome 20: R1L16—16mer, R2L14—14mer, R3L14—14mer, R4L2-2mer, and R5L8—8mer (Gao *et al.* 2023). In comparison to HORs identified using the GRMhor algorithm and the NTRPrism algorithm, the 26mer, 11mer, and 18mer HORs, as well as another version of the 8mer HOR, are missing. Additionally, 14mer and 2mer HORs appear. By comparing HOR regions and copy numbers, we can conclude that two versions of the 14mer HOR identified by HiCAT correspond to the 18mer and 26mer HORs, respectively, while the 2mer HOR corresponds to variant substructures of the Cascading 8mer HOR (see Supplementary Fig. S4).

These two comparisons with the latest tools clearly highlight the precision and thoroughness of the GRMhor algorithm, enabling it to effortlessly detect all types of HORs, irrespective of their divergence (variant HORs) or the number of monomer repetitions within a single HOR unit (Cascading HORs).

Furthermore, the GRMhor algorithm has demonstrated robust performance in identifying short tandem repeats, particularly HORs consisting of only three monomers, as illustrated in our previous studies investigating the Neuroblastoma Break Point Family genes (NBPF) (Gluncic *et al.* 2023a, 2023b). Specifically, we focused on the ∼1.6 kb NBPF repeat units, which are unique to humans and correlate with cognitive capacity in higher primates (human, chimpanzee, gorilla, and orangutan). Significantly, beyond the recognized increase in NBPF monomer copy numbers in humans compared to great apes, there are also substantial structural differences and variations in the copy number of canonical NBPF 3mer HORs between humans and their great ape counterparts. By employing the GRMhor algorithm, we successfully identified these short HORs, consisting of only three monomers, in the fully sequenced genomes of these primates. Additionally, this analysis further demonstrates that the algorithm is versatile and applicable to a wide range of repetitive units beyond just alpha satellite monomers.

Moreover, the performance of the GRMhor algorithm in identifying highly divergent HOR units is exemplified in Supplementary Figs. S5, S11, and S16 (18mer HOR in region E) and our recent article (Gluncic *et al.* 2024). In that article, we report a significantly divergent HOR array composed of 21mers, with each 21mer consisting of 21 distinct monomer types. Although this HOR array is highly divergent, lacking the presence of a canonical HOR copy, we observed some corresponding rare points in MD graph at period 21, indicating the algorithm’s capability to identify and analyse complex, divergent sequences.

It is well established that human genomes are remarkably similar, with approximately 99.9% of the genome being identical between any two individuals (Lander *et al.* 2001). This leaves only about 0.1% to account for the genetic variation that contributes to individual differences. Although we identified the same HORs in all three individual chromosome 20 genomes, the GRMhor algorithm facilitates a detailed analysis of their differences and enables comparison in terms of the number of canonical and variant copies, as well as the structure and form of the HORs themselves. As shown in the above analysis, the differences in the centromeric and pericentromeric organization of alpha satellites among the three observed individual genomes are significantly greater than 0.1%. The structures of individual HORs differ from one another, the number of HOR units in specific arrays varies (sometimes exceeding 100%), and the proportions of canonical and variant HORs differ across the arrays of individual genomes. Therefore, GRMhor is particularly suitable for studying specificities in individual genomes associated with any repetitive monomers, especially those involved in the structure of the centromere, which is crucial for the attachment of microtubules to the kinetochore and the proper segregation of genetic material during cell division.

Similarly, it is well established that humans and chimpanzees share approximately 99% of their directly comparable DNA sequences, with this similarity decreasing slightly to around 96% when accounting for DNA insertions and deletions (Chimpanzee and Analysis 2005). In this study, we have demonstrated that the centromeric region of chromosome 20 exhibits significantly greater differences than those previously reported. Although the number of alpha satellite building blocks is smaller, these monomers in humans are organized into much larger higher-order structures. In contrast, in other primates, alpha satellite monomers are predominantly organized into very long arrays of 2mer or 4mer HORs. which differ significantly from the alpha satellite HORs of higher primates in terms of composition, structure, and both total and canonical unit numbers. This example highlights the additional utility of the GRMhor algorithm as a valuable tool for the detailed study of centromere evolution, as well as any genomic region composed of repetitive units, particularly gene-rich regions such as the NBPF gene family (Gluncic *et al.* 2023a, 2023b).

Finally, our findings underscore a noteworthy concordance between bioinformatic analyses and traditional molecular methodologies, with significant implications for the field of bioinformatics. Specifically, the identification of various bands using Southern blotting, employing satellite monomers as probes, closely parallels our bioinformatic discovery of major HOR structures featuring varying numbers of monomers. This alignment not only validates the robustness of bioinformatic approaches but also underscores their compatibility and complementarity with conventional molecular techniques. By bridging these methodologies, our study not only enhances our comprehension of genomic structures but also underscores the importance of integrating diverse scientific approaches to unravel complex biological phenomena.

### 4.1Performance evaluation, limitations, and future directions

The GRMhor algorithm is designed to work with both complete genomes and those containing assembly gaps, demonstrating flexibility in handling various genomic data. However, if the original biological DNA sequence contains the desired monomers within regions of assembly gaps—whether represented as long strings of “N”s or simply missing portions of the genome—these monomers will not be detected and will therefore be absent from the final analysis conducted using the GRMhor algorithm. Monomer extraction is performed using the MonFinder algorithm (for more details, see Section 2.2), which is robust against sequencing gaps. These gaps, represented as strings of “N”s, are not recognized as valid monomers, allowing for accurate identification through precise pairwise sequence alignment. It is also worth noting that other tools, such as BLASTN, could serve as alternatives.

On the other hand, regarding sequencing artifacts, the GRMhor algorithm does not inherently filter these out, as it processes pre-extracted monomer sequences. Therefore, we emphasize the necessity of high-quality input data and suggest employing upstream strategies to mitigate potential artifacts. These measures ensure the integrity of the HOR structures reported in our analysis, enhancing confidence in the results presented.

We conducted a runtime comparison of GRMhor and NTRprism on human chromosome 20 (T2T-CHM13 assembly) using a MacBook Pro with an Apple M3 Max chip and 36 GB memory. GRMhor completed its analysis in approximately 35 min with a computational complexity of *O*(*n*^2^), while NTRprism completed its heat map analysis in 125 s. However, the performance of NTRprism was highly sensitive to parameter settings, with smaller bin sizes resulting in significantly longer runtimes. Ultimately, NTRprism achieved better runtime efficiency compared to GRMhor. While GRMhor’s longer runtime is a noted limitation, it enables detection of all HOR structures in a straightforward manner and provides a comprehensive structural map of identified HOR sequences.

To potentially reduce runtime, a feasible approach could be to limit the alignment scope by first grouping spatially proximate monomers (directly adjacent tandems) into blocks, thereby focusing alignment within each block. This would certainly shorten processing time, but it could omit valuable overlap information between monomers across different HOR arrays, which is currently available in the overall HOR map produced by the GRMhor algorithm. Further optimization efforts are under consideration to balance runtime efficiency with structural resolution.

Finally, the overall GRM map produced by the GRMhor algorithm may become partially unwieldy when dealing with very long genomic sequences, such as entire chromosomes that can contain over 30 000 monomers. As a result, the final map can be quite lengthy and may not be suitable for displaying individual blocks of HOR sequences. Therefore, we recommend, after an initial pass through the entire chromosome and detection of separate blocks of *n*mer HOR sequences, to repeat the procedure on the isolated blocks of monomers to obtain clearer and more visually appealing representations.

## Author contributions

Matko Glunčić (Conceptualization [equal], Data curation [equal], Formal analysis [equal], Funding acquisition [equal], Investigation [equal], Methodology [equal], Project administration [equal], Visualization [equal], Writing—original draft [equal], Writing—review & editing [equal]), Domjan Barić (Data curation [equal], Formal analysis [equal], Investigation [equal], Methodology [equal], Software [equal], Validation [equal], Writing—review & editing [equal]), and Vladimir Paar (Conceptualization [equal], Supervision [lead], Validation [supporting], Writing—original draft [equal], Writing—review & editing [equal])

## Supplementary data

Supplementary data are available at *Bioinformatics Advances* online.

## Conflict of interest

All authors of this article declare that they have no conflicts of interest.

## Funding

This work was supported by the project “Implementation of cutting-edge research and its application as part of the Scientific Center of Excellence for Quantum and Complex Systems, and Representations of Lie Algebras”, grant number PK.1.1.02, European Union, European Regional Development Fund and by the Croatian Science Foundation, grant number IP-2019–04-2757.

## Data availability

The MonFinder and GRMhor (python applications) are freely available at github.com/gluncic/GRM2023. All artificial monomer arrays are available for testing on github.com/gluncic/GRM2023/tree/master/data. Reference genome sequences used to test the application are freely available at the National Center for Biotechnology Information official website:

T2T_CHM13v2.0 https://www.ncbi.nlm.nih.gov/datasets/genome/GCF_009914755.1/,

HG002v1.0.1 https://www.ncbi.nlm.nih.gov/datasets/genome/GCA_018852615.2/, HG01243v3.0 https://www.ncbi.nlm.nih.gov/datasets/genome/GCA_018873775.2/,

NHGRI_mPanTro3-v2.0_pri https://www.ncbi.nlm.nih.gov/datasets/genome/GCF_028858775.2/,

NHGRI_mGorGor1-v2.0_pri https://www.ncbi.nlm.nih.gov/datasets/genome/GCF_029281585.2/,

NHGRI_mPonAbe1-v2.0_pri https://www.ncbi.nlm.nih.gov/datasets/genome/GCF_028885655.2/.

## Supplementary Material

vbae191_Supplementary_Data
