# Supplementary material for: Efficient genome monomer higher-order structure annotation and identification using the GRMhor algorithm
Source: Bioinform Adv. 2024 Nov 28;4(1):vbae191. doi: 10.1093/bioadv/vbae191 (PMC11630843; doi:10.1093/bioadv/vbae191)

Fig. S8. Cascading 16mer alpha satellite HOR alignment. Start position 27,139,263 bp and end position 29,974,303 bp in GCA_018852615.2 chromosome 20. The numbers on the left side indicate the starting position of the first monomer in each row of the HOR copies. Each HOR unit in the HOR array is represented on the left side by a single rectangle. Rectangles with the same color represent identical HOR structures. The color legend is provided on the left side, with each color corresponding to a specific HOR structure.

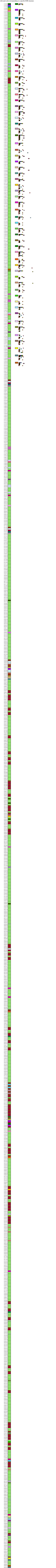

Supplement: vbae191_Supplementary_Data [file vbae191_supplementary_data.zip › FigS8.pdf]
