# Supplementary material for: Efficient genome monomer higher-order structure annotation and identification using the GRMhor algorithm
Source: Bioinform Adv. 2024 Nov 28;4(1):vbae191. doi: 10.1093/bioadv/vbae191 (PMC11630843; doi:10.1093/bioadv/vbae191)

Fig. S10. Cascading 8mer alpha satellite HOR alignment. Start position 31,274,588 bp and end position 31,364,663 bp in GCA_018852615.2 chromosome 20. The numbers on t he left side indicate the starting position of the first monomer in each row of the HOR copies.Each HOR unit in the HOR array is represented on the left side by a single rectangle. Rectangles with the same color represent identical HOR structures. The color legend is provided on the left side, with each color corresponding to a specific HOR structure.

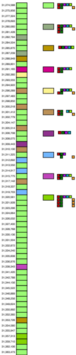

Supplement: vbae191_Supplementary_Data [file vbae191_supplementary_data.zip › FigS10.pdf]
