# Supplementary figures and images for: Efficient genome monomer higher-order structure annotation and identification using the GRMhor algorithm
Source: Bioinform Adv. 2024 Nov 28;4(1):vbae191. doi: 10.1093/bioadv/vbae191 (PMC11630843; doi:10.1093/bioadv/vbae191)

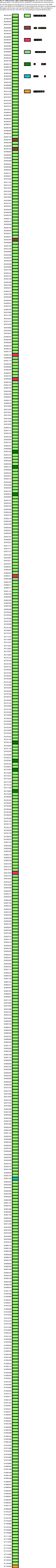

Supplement: vbae191_Supplementary_Data [file vbae191_supplementary_data.zip › FigS7.pdf]

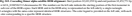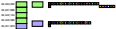

Supplement: vbae191_Supplementary_Data [file vbae191_supplementary_data.zip › FigS12.pdf]

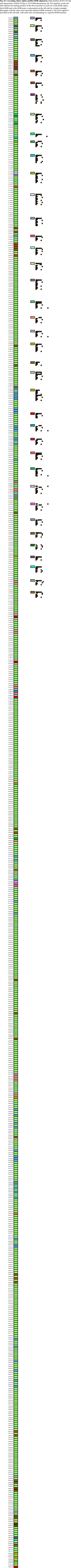

Supplement: vbae191_Supplementary_Data [file vbae191_supplementary_data.zip › FigS2.pdf]

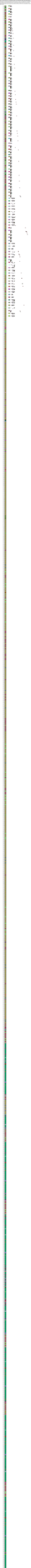

Supplement: vbae191_Supplementary_Data [file vbae191_supplementary_data.zip › FigS14.pdf]
